# Cognitive Function in Adults with Enduring Anorexia Nervosa

**DOI:** 10.3390/nu13030859

**Published:** 2021-03-05

**Authors:** Maria Seidel, Helen Brooker, Kamilla Lauenborg, Keith Wesnes, Magnus Sjögren

**Affiliations:** 1 Department of Psychological Medicine and Developmental Neuroscience, Medical Faculty, TU Dresden, 01069 Dresden, Germany; 2 Department of Medical Epidemiology and Biostatistics, Karolinska Institute Stockholm, 17165 Stockholm, Sweden; 3 Department of Psychology, Northumbria University, Newcastle NE1 8ST, UK; helen@ecogpro.com; 4 Institute for Clinical Medicine Copenhagen University, 2200 Copenhagen, Denmark; kamilla.julie.lauenborg@regionh.dk (K.L.); jan.magnus.sjoegren@regionh.dk (M.S.); 5 Medical School, Exeter University Medical School, Exeter EX1 2HZ, UK; keith@wesnes.com; 6 Wesnes Cognition Ltd., Streatley RG8 9RD, UK; 7 Centre for Human Psychopharmacology, Swinburne University, Melbourne 3122, Australia; 8 Psychiatric Center Ballerup, 2750 Ballerup, Denmark

**Keywords:** anorexia nervosa, cognitive performance, cognitive functioning, eating disorders, neuropsychology

## Abstract

Anorexia Nervosa (AN) is a severe and often enduring disorder characterized by restriction of food intake, low body weight, fear of weight gain, and distorted body image. Investigations on cognition performance in AN patients have yielded conflicting results. Using an established and sensitive computerized cognitive test battery, we aimed to assess core aspects of cognitive function, including attention span, information processing, reasoning, working and episodic memory, in AN patients and controls. Patients were recruited from the Danish Prospective Longitudinal all-comer inclusion study in Eating Disorders (PROLED). Included were 26 individuals with AN and 36 healthy volunteers (HV). All were tested with CogTrack (an online cognitive assessment system) at baseline, and AN patients were tested again at a follow-up time point after weight increase (*n* = 13). At baseline, AN patients showed faster reaction times in the attention tasks, as well as increased accuracy in grammatical reasoning compared to HV. There were no differences in cognitive function between AN patients and HV in the other cognitive domains measured (sustained attention, working and episodic memory, speed of retrieval, and speed of grammatical reasoning). No differences were visible in the AN sample between baseline and follow-up. Performance did not correlate with any clinical variables in the AN sample. These findings supplement results from other studies suggesting increased concentration and reasoning accuracy in patients suffering from AN, who showed increased performance in cognitive tasks despite their illness.

## 1. Introduction

Anorexia Nervosa (AN) is characterized by restriction of food intake (often paired with increased energy expenditure) and fear of weight gain despite being underweight, as well as a distorted body image [1]. Genetic studies strongly suggest that AN has both psychiatric and metabolic components [2]. The prevalence of AN is approximately 0.9% in women and 0.3% in men [3,4]. Treatment is usually given for long periods of time, and recovery is slow, incomplete, and accompanied by a high risk of relapse [5]. Life-threatening medical complications are known to occur during the course of the disease if not treated, contributing to a higher risk of suicide and associated death rates, corresponding to approximately 7% of those linked to psychiatric disorders [6,7].

In light of the unknown etiology and the high morbidity and mortality rates of AN patients, a better understanding of the disorder and its psychopathology is warranted, especially in order to improve existing treatment approaches. Neuropsychology has been proposed as the mediator between underlying neurobiology and psychological functioning and thereby presents an excellent opportunity for behavioral research in order to gain a deeper understanding of both the mechanisms underlying eating disorders’ (ED) behavior [8] as well as when and how to treat patients with AN [9].

Neuropsychological investigations in AN patients have revealed heterogeneous findings regarding their performance in different cognitive domains [10,11,12]. For example, while some have shown reduced performance in attentional functions (vigilance and selective attention), memory (verbal recall accuracy and speed), and executive functions (cognitive flexibility, central coherence, and decision-making) [11,13,14], others have shown only selective impairments, no differences, or even better performance compared to control groups [15,16,17,18]. However, the published studies are very heterogeneous regarding the type of cognitive tests used, how many and which domains of cognitive function were covered, and which age groups were included, warranting more standardized measures. In adult patients with AN, only a few studies with large sample sizes have been conducted, using a variety of cognitive tests and test batteries. Results report a trend towards no differences or decreased performance for AN patients compared to healthy volunteers (HV) in the speed of processing, working memory, attention, and set shifting [11,17,19,20,21,22,23]. Additionally, Stedal and colleagues [24] found decreased set-shifting and memory capacity, but increased verbal fluency in patients with AN, when using the Ravello profile. Some studies have described normalization or a trend towards normalization after weight restoration of different cognitive domains, especially in younger samples [18,20,25,26], while others have reported persisting alterations even in the recovered state [27,28,29] or inconclusive findings for adult patients [30]. Similarly, findings regarding associations between clinical variables such as body mass index (BMI) and ED symptoms and cognitive functioning appear to be heterogeneous [31,32,33,34,35]. Clarifying the extent of a potential cognitive impairment in moderately and severely underweight AN patients is important, since this may counteract and interfere with treatment attempts and may be an outcome for clinical assessments.

In the past, most studies investigating eating disorders and associated cognitive performance have used traditional paper-and-pencil cognitive tests [13], which have an extensive literature base. However, over the last 35 years, recognition has increased for computerized cognitive tests that allow the presentation of tests and stimuli to be more standardized between participants. These tests remove problems with interrater reliability and add the possibility of measuring millisecond reaction times that are essential to establishing processing and decision-making time, therefore enabling the reliable detection of differences. Additionally, the tests permit repeated testing with the ease of parallel form use. However, it is evident from the eating disorder literature that there are only a few studies that have utilized computerized cognitive test batteries, including the CANTAB [36,37], the Cognitive Drug Research (CDR) system [31,38,39], the CogState battery, and the IntegNeuro cognitive battery [18]. The results have been conflicting, ranging from subtle changes [36] that normalized with increasing weight [18] to changes in motor inhibition [31,37] and attention [31]. 

To assess major aspects of cognitive function relevant to everyday life in adult patients with AN, we used the computerized cognitive test battery CogTrack^TM^, including tests of attention, information processing, reasoning, working and episodic memory. AN participants at baseline and follow-up as well as HV were part of the Prospective Longitudinal All-comer inclusion study on Eating Disorders (PROLED). Based on previous findings regarding deficits in cognitive functioning in acute as well as recovered AN patients, we expected decreased performance of patients with AN compared to HV, as well as an increase in performance at the follow-up time point after weight gain. We further assumed clinical variables presenting ED severity (ED symptoms and BMI) to correlate with cognitive performance in the AN sample during the acute stage.

## 2. Materials and Methods

### 2.1. Participants

Between January 2016 and December 2017, 26 (24 female) adult patients (28.62 years ±9.7) diagnosed with AN, together with 36 HV (30 female; 25.42 ± 3.42 years), participated in the PROLED study. The ongoing PROLED study is a clinical naturalistic 10-year annual follow-up study. The PROLED study has been reviewed and approved by the National Committee on Health Research Ethics (Ethical Application Id: DNVK Journal no: H-15012537) and is conducted in compliance with the Declaration of Helsinki and International Conference on Harmonisation and Good Clinical Practice Guidelines. All subjects participating in the PROLED study provided their written informed consent. 

Inclusion criteria for the patient group was an AN diagnosis according to ICD-10 [40] (50.0 or 50.1), age >18 years, and informed consent. All patients were recruited through their referral to the Psychiatric Center Ballerup (PCB). All patients were in various stages of a weight restoration program, as either inpatients or in intensive day-care treatment (4 weekdays out of 5). Meals were provided 3 (daycare) to 5 (inpatient) times per day, and food intake was monitored by trained nurses. A dietician had planned the meals individually to enable a 1 kg weight gain per week. All patients had undergone medical and psychiatric examinations, and any medical complications had been addressed. Comorbid diagnoses as well as medication were derived from medical records. Of the 26 individuals, 16 received psychotropic medication, i.e., antidepressants (*n* = 10) and/or antipsychotic medication (*n* = 9). Nineteen individuals were diagnosed with one or more comorbid disorders, the most common being neurotic, stress-related, and somatoform disorders (F40.0–48; *n* = 12), mood disorders (F30.0–39; *n* = 9), and disorders of personality and behavior in adult individuals (F60.0–69; *n* = 4). None of the patients was on systematically provided psychotherapy at the time of assessment, while some had infrequent, non-regular, supportive dialogues with psychologists. Subjects undergoing forced care were excluded from the study. A subsample of patients with AN (*n* = 13) were also assessed before discharge (between baseline and follow-up measurement, which was on average after 100 days; BMI increased, on average, by 2.01). 

HV were recruited via public advertisement. They had to be of normal weight (BMI > 18.5 or BMI < 25) and between 18 and 45 years old. Exclusion criteria were a lifetime psychiatric diagnosis, medication intake during the last 3 months, abnormal eating behaviors, as well as any chronic illnesses. 

The patients and controls were asked to detail the highest level of education completed, ranging from (1) Secondary Education (GCSE/O-Levels) to (7) Higher University training, indicating the years spent in education. We estimated the sample size based upon previous studies using similar cognition instruments [39].

### 2.2. Clinical Measures

Initial diagnostic assessments were made using the Psychiatric State Examination (PSE) diagnostic interview [41], Eating Disorder Examination (EDE diagnostic questions [42]), and routine clinical and laboratory assessments for the diagnosis of AN and comorbid disorders. The PROLED study includes validated questionnaires that assess general and specific aspects of ED, as well as co-morbid disorders such as depression, anxiety, personality dysfunctions, previous traumas, autism, cognition, social aspects, global function, quality of life, and psychotic features. A number of questionnaires are used in the PROLED study, and for this particular investigation, the Major Depressive Inventory (MDI; [43]), the Eating Disorder Examination-Questionnaire (EDE-Q; [44]), and the Global Assessment of Functioning (GAF-F; [45]) were used. 

The EDE-Q was administered as a questionnaire, and the scores for the 4 subscales (Restraint, Eating concern, Shape concern, and Weight concern subscales) as well as the global score, were assessed. The MDI was also administered as a questionnaire, and the total scores were collected. GAF-F was assessed by the primary investigator (JMC) in accordance with the procedure described in DSM-IV [46]. Weight was assessed weekly using a calibrated medical weighing scale by a trained nurse, and BMI was calculated as kg/m^2^.

### 2.3. Cognitive Assessments

The CogTrack^TM^ system is an internet-based set of cognitive tests, which assess major aspects of cognition including attention, information processing, reasoning, working and episodic memory [47,48]. For reliability and normative values of the test battery, please refer to Wesnes et al. [49]. The 11 tasks take just over 20 minutes to be performed and are listed in the order in which they were assessed (see Supplementary Material (1.1)). Tasks include assessments of Immediate Word Recall, Pattern Separation (part 1 and 2), Simple Reaction Time, Digit Vigilance, Choice Reaction Time, Spatial Working Memory, Numeric Working Memory, Delayed Word Recall, Word Recognition, and Grammatical Reasoning. Tasks were shown in a standardized form to all participants on a study laptop in a quiet environment. For a detailed description of the tasks, please refer to the Supplementary Material (1.1). 

Training effects in cognitive testing have long been established [50,51]; in order to overcome this, both patients and controls were required to perform all CogTrack tests on two occasions on a separate day prior to baseline. This procedure allows patients and controls to familiarize themselves with the cognitive tests in order to establish a valid baseline once they have understood what to expect from the tests. The conditions of the training and the baseline and follow-up sessions are identical, providing follow-up results that are not biased by learning effects. The training data were only recorded but not analyzed. 

### 2.4. Statistical Analyses

#### Calculation of Composite Scores

Composite outcome scores from the CogTrack tasks were, as previously described, based on the established factor structure of the cognitive tasks [48]. From the three attention tasks, (Simple Reaction Time, Digit Vigilance, and Choice Reaction Time), two composite scores were created: the Attentional Intensity Index, which takes the sum of the three reaction time scores, and the Sustained Attention Index, which combines the three accuracy scores. The Working Memory Capacity Index includes the accuracy scores from the spatial and numeric working memory tasks, and the Episodic Memory Capacity Index merges the accuracy scores from the Pattern Separation, Word Recognition, and immediate and delayed Word Recall tasks, whilst also considering the number of errors. The reaction time scores for Numeric Working Memory, Spatial Working Memory, Word Recognition, and Pattern Separation are combined to create the Speed of Retrieval Index. The Grammatical Reasoning task produces two outcome measures: overall accuracy and overall speed.

All analyses were conducted using the SPSS Version 26. BMI, age, and education were compared between groups using independent samples t-tests. All tests were 2-tailed, with an alpha level of 0.05. For the cross-sectional analysis, ANCOVAs were conducted comparing the two groups at baseline, fitting covariates for age, gender, and education. In case of non-normal distribution of composite scores, ranked ANCOVA [52] was calculated to ensure the validity of the results. For the follow-up analyses, repeated measures ANCOVAs were conducted on composite scores of the patients with AN, comparing the baseline session to the follow-up session prior to discharge, fitting covariates for age, gender, and education. As sample size was low, we also reported effect sizes for the results of the ANCOVAS (partial eta-squared (partial η^2^). Partial η^2^ values of 0.01, 0.06, and 0.14 were considered as benchmarks for small, medium, and large effect sizes, respectively [53]. Correlations between cognitive function and various clinical characteristics of the patients were performed using Pearson’s correlation (r) or Spearman’s rank correlations (rho) if the data were not normally distributed. 

## 3. Results

### 3.1. Sample

The descriptive data in the sample are summarized in Table 1 (for descriptives separated by gender, see Supplementary Material Table S1). Patients had a significantly lower level of education (*p* < 0.01) which is why education was added as covariateto all group comparisons. 

### 3.2. Cognitive Functioning

There was a significant difference between AN and HV in the Attentional Intensity Index, which reflects the participants’ intensity of concentration, as well as in Grammatical Reasoning overall accuracy, which reflects the ability to accurately reason about statements that may or may not be true (Table 2). Both effects were of medium size (partial η^2^). There were no significant differences between patients with AN and HV on any of the other cognitive composite measures at baseline (Table 2). For results of group comparisons between the single tasks of the Cogtrack battery, please see Supplementary Material Table S2. Additional analysis investigating ranked ANCOVAS for outcome variables without normal distribution [52] did not result in a change of significance (Supplementary Material Table S3). Further, excluding male participants from the analysis did not change effect sizes in the group comparisons of the Attentional Intensity Index and Grammatical Reasoning Accuracy score (Supplementary Material Table S4). For the subsample of patients with AN providing baseline and follow-up data, results showed no differences in the composite scores between the two time points (Supplementary Material Table S5).

We identified no significant correlations between the composite scores of the cognitive tasks and clinical or psychometric variables (BMI, ED symptoms, illness duration, age of onset) in the AN sample (Supplementary Material Table S6). 

## 4. Discussion

The current study investigating differences in cognitive performance (including attention, information processing, reasoning, working and episodic memory) between AN patients during inpatient or day care and HV using an established cognitive test battery (CogTrack). We found differences between the groups in their ability to concentrate and reason accurately. During the first assessment, individuals diagnosed with AN responded faster in the attention tasks, showing superior concentration and increased accuracy when reasoning about grammatical statements compared to HV. Additionally, cognitive performance in adult patients with AN was at the same level for the other sub-scores of the battery (e.g., sustained attention, episodic and working memory capacity, retrieval speed, and grammatical reasoning reaction time). Performance in the AN sample was not correlated with BMI or duration of the illness. Thus, in spite of using a sensitive test battery assessing core aspects of cognitive function, we could not detect deficits in the assessed domains in this adult AN sample compared to HV. This is even more astonishing, as the duration of the illness and the number of comorbidities indicated that the sample consisted of chronic and severely ill patients. Moreover, increased performance in AN patients on two aspects of cognitive function, namely, concentration (attentional intensity) and reasoning (accuracy), were found. While this is contrary to previous findings suggesting deficits in cognitive functioning (e.g., executive functions such as set-shifting or central coherence) in acute patients with AN [11,54,55], the reported findings are in line with clinical observations of increased perfectionism and overachievement in this population [56], which might even contribute to reduced central coherence and set shifting by using too many resources for one task, instead of allocating them flexibly to other relevant stimuli. The Grammatical Reasoning Accuracy score represents the participants´ ability to reason about statements referred to differing images, which is generally considered an ability linked to problem-solving and concentration and working memory capacities [57,58]. A speculative interpretation might be that the increased accuracy of AN patients in this sub-score could also be linked to increased cognitive processes allocated to problem-solving, which could be related to characteristics such as increased rumination, that is often found in this patient population [59,60]. Both findings contribute to experimental and neural evidence hinting at elevated cognitive control mechanisms in AN patients [61,62,63], as well as increased intelligence quotient (IQ) [64], which might be able to explain the better performance of these patients, despite their illness. Unchanged levels of cognitive functioning in the other subscales (sustained attention, working and episodic memory, speed of retrieval, and speed of grammatical reasoning) resemble the results of previous research that has found no differences between AN patients and HV in cognitive functioning, albeit the use of different computerized cognition tests [18,31,36,37]. The absence of an association between cognitive functioning and BMI and ED symptoms in the examined sample is in line with previous research reporting little or no effect on test performance in patients with AN before and after therapy [65,66,67]. Cognitive performance in this patient sample with enduring AN and a very low BMI, similar to that of HV, is even more astonishing considering the severe atrophic changes found during the acute state [68,69]. Increased or unchanged scores in performance might reflect the brain´s extensive compensatory mechanisms to adapt to extreme conditions. The implications of this may be that treatment regimens could be modified, e.g., that psychotherapy may be initiated when patients are still in an early phases of recovery. 

The current study has to be seen in the light of the following limitations. Firstly, only individuals with AN and a BMI of 14 or above where eligible to participate in the study, and thereby, cognitive performance in individuals with a BMI below 14 using the CogTrack tests is unknown. Secondly, due to the long duration of illness in some patients, as well as the high frequency of comorbidities in the examined sample, patients had to be enrolled whenever they were eligible and accepted to undergo cognitive testing, which meant they were at different stages of their treatment program, which might have influenced the results. However, we could not find a correlation between cognitive performance and BMI, which may speak against an impact of BMI (or stage of weight restoration) on the domains of cognitive performance measured in the study. Thirdly, including male participants, as well as not applying pairwise matching for age and education between AN and HV, might have brought some heterogeneity to the study sample that could not be completely addressed by adding these variables as covariates to the analyses. We are aware of these shortcomings and encourage further research with samples matched for the above-mentioned characteristics. Another limitation was the short and heterogeneous time to follow-up for some patients, which again reflected the severity and complexity of this group of patients, who were subjected to sudden changes in their clinical course. This factor may limit the validity of our findings for changes over time and should be explored further. Finally, we acknowledge that there are studies including larger samples of individuals for cognitive testing, and although the sensitivity of the CogTrack reduces variability in factors related to methodology and testing, it is encouraged to increase the sample size. 

Despite the mentioned limitations, the possibility to accurately measure core aspects of cognitive function in this population has beenverified. The employment of a computerized cognitive test battery allowed measuring millisecond reaction times, which enabled the capturing of performance trade-offs between speed and accuracy in core measures of attention, vigilance, episodic memory, working memory, and reasoning. Another strength of the study is the use of repeated practice sessions before the baseline measure to manage practice effects for the assessment at the second time point, which otherwise could influence the results and lead to erroneous conclusions [50]. Further, the assessment of cognitive function in general is affected by the use of many different tests to assess single domains of cognitive function as well as of different methodological setups, which, together, make comparisons of the results of different studies very difficult. Applying computerized, standardized, and validated test batteries would enable researchers to better compare differences and results between different studies as well as patient populations or nationalities.

## 5. Conclusions

The current study finds that the Sustained Attention score and Grammatical Reasoning Accuracy were better in patients with AN, while no differences between AN patients and HV in the other composite scores of the CogTrack test battery were identified. These findings might suggest that behavioral interventions might be initiated already in early phases of treatment of AN. Computerized cognitive assessments have demonstrated to be a well-received and reliable tool for investigating cognition in patients with AN.

## Figures and Tables

**Table 1:** Result of independent samples t-test comparing descriptive statistics between patients with AN at baseline and HV.

Descriptive Statistics						
	AN (*N* = 26)		HV (*N* = 36)			
	Mean	SD	Mean	SD	t	*p*
Age	28.62	9.70	25.42	3.42	1.61	>0.05
BMI	17.31	1.89	23.14	2.9	−8.92	<0.001
Age of onset	22.72	7.69				
Duration of Illness	9.19	5.35				
EDE-Q	3.08	1.60	0.86	0.95	6.31	<0.001
GAF	46.00	12.34	99.46	2.83	−20.34	<0.001
Education	4.08	0.27	5.28	1.39	−2.83	<0.01

AN = anorexia nervosa; HV = healthy volunteer; SD = standard deviation; BMI = body mass index (calculated as kg/m^2^). Duration of illness is given in years and defines the period from the first episode to the research date; EDE-Q = Eating Disorder Examination Questionnaire version; GAF = Global Functioning; Education is rated 1 (lowest) to 7 (highest).

**Table 2:** Results of ANCOVA, adjusting for age, education, and gender, comparing results of composite scores between patients with AN at baseline and HV, displaying mean, standard deviation, *p*-values, and effect sizes. Significant differences were highlighted in bold.

Group Comparisons of Composite Scores						
Composite Scores	HV (*n* = 36)	SD	AN (*n* = 26)	SD	*p*	η^2^
Attentional Intensity Index	1241.50	119.15	1203.60	136.58	**0.040**	**0.07**
Sustained Attention Index	90.56	4.72	91.21	5.84	0.119	0.04
Quality of Memory	364.55	71.29	360.75	64.29	0.581	0.00
Working Memory Capacity Index	92.37	5.96	91.56	6.71	0625	0.00
Episodic Memory Capacity Index	152.42	52.76	154.39	40.55	0.679	0.00
Speed of Retrieval Index	2359.02	360.74	2485.94	296.28	0.340	0.02
Grammatical Reasoning Overall Accuracy	83.42	15.07	90.27	9.65	**0.025**	**0.09**
Grammatical Reasoning Overall Speed	2845.38	922.09	2677.64	649.47	0.225	0.03

η^2^ = partial eta squared.

## Data Availability

The data are available for re-analyses per request, and the study is registered in clinical trials.gov: NCT03224091.

## Supplementary Materials

The following are available online at https://www.mdpi.com/2072-6643/13/3/859/s1, Material (1.1); Table S1: Descriptive Statistics separated by gender, Table S2: Group comparisons of single tasks scores using analysis of variance with age, education and gender included as covariates (ANCOVA), Table S3: Group comparisons of non-normally distributed composite scores using ranked ANCOVA; Table S4: Group comparisons using analysis of variance with age, education and gender included as covariates (ANCOVA) excluding male participants, Table S5: Longitudinal analyses of BMI using a dependent sample t-test as well as longitudinal analysis of composite scores using analysis of variance with age, education and gender included as covariates (ANCOVA) in the AN sample only, Table S6: Associations between cognitive function and clinical variables.
