# Supplementary material for: Cognitive Function in Adults with Enduring Anorexia Nervosa
Source: Nutrients. 2021 Mar 5;13(3):859. doi: 10.3390/nu13030859 (PMC7998517; doi:10.3390/nu13030859)
Supplement: Supplementary file 1 [file nutrients-13-00859-s001.pdf]

Supplementary Material, Seidel et al.

1.1. Material

For detailed description of the single tasks please see Wesnes et al.[1] or the list below.

1) Immediate Word Recall: A list of 15 words is presented on the screen, at the rate of 1 word every 2 seconds, for the subject to remember. The subject is then given one minute to type all words that can be recalled, in any order, using the computer keyboard.
2) Pattern Separation: In this first stage, a series of 20 pictures of everyday scenes and objects is presented on the screen at the rate of 1 picture every 3 seconds for the subject to remember. The subject is instructed to just view the images which will all be reshown later, mixed together with very similar ones.
3) Simple Reaction Time: The subject is instructed to press the right arrow key on the keyboard as quickly as possible every time a stimulus is presented in the centre of the screen (the inter-stimulus interval varies randomly between 1 and 3.5 seconds). The subject is informed that fifty stimuli will be presented one at a time and that the stimuli will remain there until a response is made.
4) Digit Vigilance: A target digit from 1 to 9 is randomly selected and constantly displayed to the right hand side of the screen. Digits are then presented one at a time in the centre of the screen at a screen rate of 150 per minute. The subject is required to press the right arrow key as quickly as possible every time a digit matches the target digit.
5) Choice Reaction Time: The two possible stimuli in this task are either the right facing arrow with the word 'YES' in the middle, or a left facing arrow, with the word 'NO' in the middle. On each of 50 successive trials one of these two stimuli are selected randomly (but with equal probability) and presented in the centre of the screen, remaining there until a response is made. Subjects are asked to respond as quickly and accurately as possible.
6) Spatial Working Memory: A 3 by 3 array of light bulbs is presented on the screen for a 10 second period. Four of the nine light bulbs are lit. The subject must memorise the position of the lit bulbs. There are then 36 subsequent presentations of the 3 by 3 array, each time with only a single bulb lit. For each presentation, the subject is required to decide whether or not the lit bulb was one of those lit in the original presentation, pressing the right keyboard arrow if it was, and the left if it was not, as quickly and accurately as possible. The images remain on the screen until a response is made.
7) Numeric Working Memory: A series of 5 different digits is presented on the screen at the rate of 1 every 1.2 seconds. The subject is instructed to hold the digits in memory. This is followed by a series of 30 probe digits, each of which remains on the screen until a response is made. For each stimulus, the subject has to indicate whether or not it was

in the original series, pressing the right keyboard arrow if it was, and the left if it was not, as quickly and accurately as possible. Each number remains on the screen until a response is made.

8) Delayed Word Recall: The subject is given one minute to type as many of the words as can be recalled from the list presented earlier, in any order, using the computer keyboard.
9) Word Recognition: The 15 original words plus 15 distractor words are presented one at a time in a randomized order. For each word the subject is required to indicate whether or not it was from the original list of words by pressing the right keyboard arrow if it was, and the left if it was not, as quickly and accurately as possible. Each word remains on the screen until a response is made.
10) Pattern Separation: In this second stage the original pictures plus the 20 very similar distractor pictures are presented one at a time in a counterbalanced order. Half of the original pictures are presented prior to the very similar distractor versions, and half afterwards. For each picture the subject has to indicate whether or not it was the precise picture shown earlier, pressing the right keyboard arrow if it was, and the left if it was not, as quickly and accurately as possible. Each picture remains on the screen until a response is made.
11) Grammatical Reasoning: Either an image consisting of a circle contained by a square or a square contained by a circle are presented, together with a statement above describing the relationships between the shapes. The 8 different statements vary in grammatical complexity with ‘is smaller than’, ‘is not smaller than’, ‘is bigger than’, is not bigger than’, ‘contains’, ‘does not contain’, ‘is contained by’ and ‘is not contained by’. There are 32 combinations with half of the statements true and half false. The subject must decide whether each statement is true or false, pressing the right or left arrow key as appropriate, as quickly and accurately as possible. Each image remains on the screen until a response is made.

1.2. Results

Table S1: Descriptive Statistics

	**AN male (N=2)**		**AN female (n=24)**		**HV male (n=6)**		**HV female (n=30)**	
	Mean	***SD***	***Mean***	***SD***	***Mean***	***SD***	***Mean***	***SD***
**Age**	**28.5**	**3.5**	**28.63**	**10.09**	**25**	**4.8**	**25.50**	**3.12**
**BMI**	**17.6**	**0.25**	**17.29**	**1.96**	**23.21**	**2.93**	**23.12**	**2.96**
**Age of onset**	**21.5**	**2.1**	**19.25**	**10.04**				
**Duration of Illness**	**7**	**1.4**	**9.38**	**5.51**				
**EDE-Q**	**3.98**	**0.77**	**3**	**1.63**	**0.33**	**0.28**	**0.97**	**1.01**
**GAF**	**55**	**0**	**45.14**	**12.6**	**100**	**00**	**99.32**	**3.2**
**Education**	**4.5**	**3.5**	**4.04**	**1.9**	**4.83**	**2.23**	**5.37**	**1.2**

Table S1: Descriptives (mean and standard deviation) of the sample, seperated by gender. SD=Standard Deviation, AN=Anorexia Nervosa, HV=Healthy Volounteers.

Table S2:

Variable	Diagnoses	N	MEAN	SD	p-value (ANCOVA)
Immediate Word Recall - Words Correctly Recalled (%)	AN	26	43.59	14.26	n.S.
	HV	36	42.59	16.19	
Immediate Word Recall - Errors (#)	AN	26	1.58	1.42	n.S.
	HV	36	2.36	1.79	
Simple Reaction Time – Median Speed (msec)	AN	26	307.32	51.93	n.S.
	HV	36	321.34	51.33	
Digit Vigilance - Average Speed (msec)	AN	26	447.63	41.04	n.S.
	HV	36	461.81	38.81	
Digit Vigilance - Targets Detected (%)	AN	26	95.39	8.00	n.S.
	HV	36	96.30	4.84	
Digit Vigilance - False Alarms (#)	AN	26	1.96	1.51	n.S.
	HV	36	2.28	1.85	
Choice Reaction Time - Accuracy (%)	AN	26	95.69	3.70	n.S.
	HV	36	94.94	3.40	
Choice Reaction Time – Median Speed (msec)	AN	26	448.65	59.84	n.S.
	HV	36	458.34	55.69	
Spatial Working Memory Original Stimuli - Accuracy (%)	AN	26	96.39	5.91	n.S.
	HV	36	94.97	6.48	
Spatial Working Memory New Stimuli - Accuracy (%)	AN	26	98.27	3.14	n.S.
	HV	36	97.36	3.27	
Spatial Working Memory Original Stimuli - Average Speed (msec)	AN	26	821.46	181.68	n.S.
	HV	36	736.95	145.36	
Spatial Working Memory New Stimuli - Average Speed (msec)	AN	26	842.62	176.99	n.S.
	HV	36	817.19	164.44	
Numeric Working Memory Original Stimuli - Accuracy (%)	AN	26	90.51	10.18	n.S.
	HV	36	94.26	6.60	
Numeric Working Memory New Stimuli - Accuracy (%)	AN	26	97.95	3.66	n.S.
	HV	36	98.15	3.42	
Numeric Working Memory Original Stimuli - Average Speed (msec)	AN	26	753.85	197.40	n.S.
	HV	36	757.63	228.97	
Numeric Working Memory New Stimuli	AN	26	796.13	138.96	n.S.
	HV	36	770.31	168.10	
Numeric Working Memory - Average Speed (msec)	AN	26	772.89	138.93	n.S.
	HV	36	764.24	184.22	

Delayed Word Recall - Words Correctly Recalled (#)	AN	26	5.04	2.11	n.S.
	HV	36	4.83	3.06	
Delayed Word Recall - Words Correctly Recalled (%)	AN	26	33.59	14.04	n.S.
	HV	36	32.22	20.38	
Delayed Word Recall - Errors (#)	AN	26	2.00	1442.00	n.S.
	HV	36	2.47	2782.00	
Word Recognition Original Stimuli - Accuracy (%)	AN	25	73.60	18.31	n.S.
	HV	36	78.89	19.49	
Word Recognition New Stimuli - Accuracy (%)	AN	25	90.13	10.39	n.S.
	HV	36	92.22	8.80	
Word Recognition Original Stimuli - Average Speed (msec)	AN	25	849.19	169.86	n.S.
	HV	36	820.20	156.73	
Word Recognition New Stimuli -Average Speed (msec)	AN	25	856.77	157.23	n.S.
	HV	36	811.63	160.67	
Word Recognition - Average Speed (msec)	AN	25	849.87	147.02	n.S.
	HV	36	813.73	139.83	
Pattern Separation Original Stimuli - Accuracy (%)	AN	26	82.12	17.10	n.S.
	HV	36	85.56	12.18	
Pattern Separation New Stimuli - Accuracy (%)	AN	26	83.65	9.44	n.S.
	HV	36	80.56	11.82	
Pattern Separation Original Stimuli - Average Speed (msec)	AN	26	1203.42	286.82	n.S.
	HV	36	1118.89	273.83	
Pattern Separation New Stimuli - Average Speed (msec)	AN	26	1148.07	220.34	n.S.
	HV	36	1126.15	247.68	
Pattern Separation - Average Speed (msec)	AN	26	1169.29	228.58	n.S.
	HV	36	1123.02	239.33	
Grammatical Reasoning - Accuracy (%)	AN	26	90.27	9.65	**0.025**
	HV	36	83.42	15.07	
Grammatical Reasoning – Median Speed (msec)	AN	26	2677.64	649.47	n.S.
	HV	36	2845.38	922.09	

Table S2: Group comparisons of single tasks scores using analysis of variance with age, education and gender included as covariates (ANCOVA). Tables display mean and standard deviations of each group. AN=Anorexia nervosa patients at baseline, HV=Healthy volunteer, n.S.=$p<0.05$

Table S3: Group comparisons of non-normally distributed composite scores using ranked ANCOVA

	HV (n=36)	SD	AN (n=26)	SD	F	p	η²
**Attentional Intensity Index**	3.67	16.90	-5.11	16.96	4.08	**0.048**	**0.06**
**Working Memory Capacity Index**	0.9	17.04	-1.25	18.75	0.22	0.643	0.00
**Grammatical Reasoning Overall Accuracy**	5.68	16.92	-4.11	16.38	5.25	**0.026**	**0.08**

Table S3: Results of ranked analysis of variance using gender, eductaion and age as covariates. Method according to Quade et al. [2]. Results displaying mean and standard deviations of the residuals of the linear regression using ranked covariates /age, gender, education). F, p and partial η^2 values remained similar to main analyes using parametric approach.

Table S4: Group comparisons excluding male participants

	HV (n=30)	SD	AN (n=24)	SD	F	p	η²
**Attentional Intensity Index**	1227.52	108.78	1203.14	142.38	3.84	**0.056**	**0.071**
**Grammatical Reasoning Overall Accuracy**	85.63	13.22	90.50	9.86	4.91	**0.031**	**0.089**

Table S4: Results of analysis of variance using eductaion and age as covariates. Results displaying mean and standard deviations, F, p values. Partial η^2 values remained similar to main analyes using full sample.

Table S5

	T1 (n=13)	SD	T2 (n=13)	SD	p
BMI	15.95	1.02	17.97	1.17	<0.001
Attentional Intensity Index	1183.18	63.72	1204.08	97.01	0.490
Sustained Attention Index	92.05	6.88	98.10	8.54	0.445
Quality of Memory	351.23	39.15	376.89	37.28	0.746
Working Memory Capacity Index	92.08	5.10	95.30	4.21	0.551
Episodic Memory Capacity Index	144.02	26.69	157.51	33.24	0.527
Speed of Retrieval Index	2450.89	285.63	2355.26	263.18	0.590
Grammatical Reasoning Overall Accuracy	90.87	8.12	93.27	4.00	0.474
Grammatical Reasoning Overall Speed	2757.02	709.00	2819.42	742.44	0.417

Table S5: Longitudinal analyses of BMI using a dependent sample t-test as well as longitudinal analysis of composite scores using analysis of variance with age, education and gender included as covariates (ANCOVA) in the AN sample only. Tables display mean and standard deviations of each group. Duration between T1 and T2 ranged from 20-337 (mean=100) days. T1=Anorexia nervosa patients at baseline, T2=Anorexia nervosa patient at follow-up before discharge, HV=Healthy volunteer. None of the comparisons was significant.

Table S6: Associations between cognitive function and clinical variables

	EDE-Q	BMI	Illness duration	Age of onset
**Attentional Intensity Index**	0.17	0.22	0.17	0.11
**Sustained Attention Index**	0.20	-0.11	-0.21	0.06
**Working Memory Capacity Index**	0.23	0.01	-0.01	-0.14
**Episodic Memory Capacity Index**	-0.34	0.27	-0.21	-0.24
**Speed of Retrieval Index**	0.14	0.16	0.24	0.25
**Quality of Memory**	-0.29	0.26	-0.21	-0.24
**Grammatical Reasoning Accurarcy**	-0.10	-0.15	-0.05	-0.07
**Grammatical Reasoning Median speed**	0.25	-0.07	0.27	0.13

Table S6: Pearson Correlation coefficients between composite scores at baseline and clinical variables BMI, EDE-Q, duration of illness in the AN group. BMI=body-mass index, EDE-Q= Eating disorder examination questionnaire version. None of the associationswas statistically significant. Significance of results remained the same when applying non-parametric Spearmans' ranked correlation (rho) for non normaly distributed data (Attentional Intensity Index, Working Memory Capacity Index, Grammatical Reasoning Accuracy, EDE-Q).

References:

1. Wesnes, K.A.; Brooker, H.; Watson, A.W.; Bal, W.; Okello, E. Effects of the Red Bull Energy Drink on Cognitive Function and Mood in Healthy Young Volunteers. *Journal of Psychopharmacology* **2017**, *31*, 211–221.
2. Quade, D. Rank Analysis of Covariance. *Journal of the American Statistical Association* **1967**, *62*, 1187–1200.
